# Experimental Quantification of the Electro-Osmotic Flow Field in an Irregular Porous Medium and Its Consequences for Dewatering

**DOI:** 10.1021/acs.iecr.6c01798

**Published:** 2026-08-28

**Authors:** Aza Alawi, Ankur D. Bordoloi, Johan T. Padding, Valeria Garbin

**Affiliations:** † Department of Chemical Engineering, 2860 Delft University of Technology, van der Maasweg 9, Delft 2629 HZ, The Netherlands; ‡ Department of Process and Energy, 2860 Delft University of Technology, Leeghwaterstraat 39, Delft 2628 CB, The Netherlands

## Abstract

The development of dewatering methods driven by electric fields offers significant potential to reduce the energy demand for these processes. Electro-osmotic dewatering relies on the application of an electric field across a porous matrix possessing a surface charge, inducing liquid transport through electrokinetic mechanisms. While the feasibility and energy efficiency of electro-osmotic dewatering have been demonstrated, a fundamental understanding of how the irregular, porous microstructure affects the uniformity of dewatering is lacking. In this work, we investigate electro-osmotic flow in porous media micromodels and directly compare it with conventional pressure-driven flow under controlled experimental conditions. The micromodel is designed and microfabricated to exhibit a controlled, heterogeneous microstructure. Pore-scale velocity fields are measured by using particle tracking velocimetry and characterized through velocity probability density functions. A multiphysics model, coupling ion transport, electrostatics, and hydrodynamics, is validated against the experiments. The results show that, in contrast to pressure-driven flow, which exhibits broader velocity distributions with preferential flow paths and stagnant regions, electro-osmotic flow produces a narrower velocity distribution, even in heterogeneous porous structures. These findings demonstrate the potential of electro-osmotic dewatering to achieve more uniform water removal in low-permeability materials and provide a predictive framework for optimizing electrically driven dewatering.

## Graphical abstract

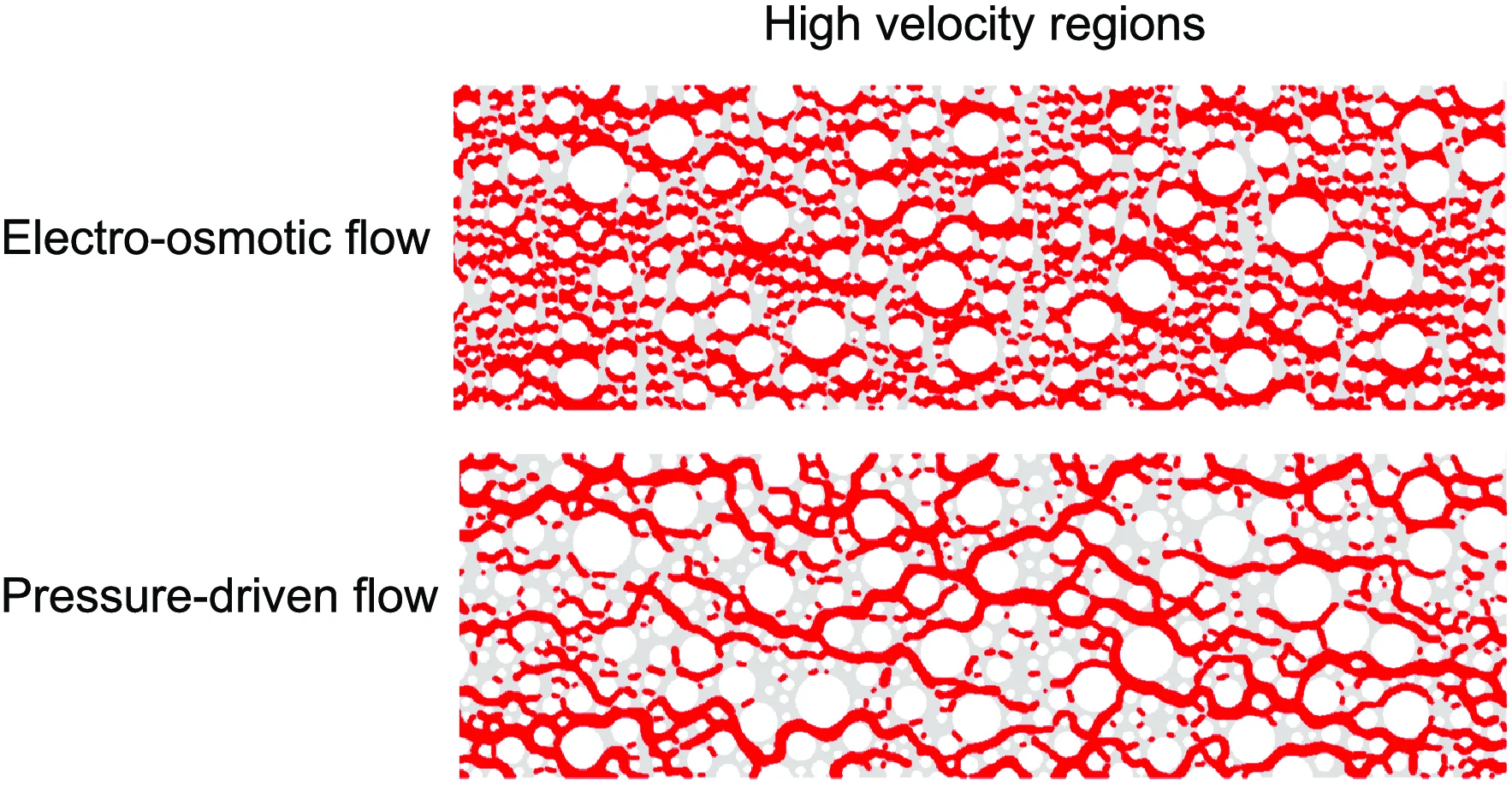

## Introduction

Dewatering—a crucial process in civil and environmental engineering and in the food and paper industry—currently relies mostly on mechanical forces. For instance, pressure-assisted dewatering is widely applied for sludge dewatering,^1^ biomass processing,^2^ food production,^3^ and paper manufacturing.^4^ Pressure-driven dewatering can be energy-intensive, particularly for low-permeability materials, where large pressure gradients are required.^5^ As part of the electrification of the process industry,^6^ electrical dewatering techniques, including electro-osmotic dewatering^7,8^ are emerging as alternative or complementary approaches for water removal.

Electro-osmotic flow occurs in porous matrices presenting a surface charge, such as soil and biomass. These matrices typically carry negative surface charges,^9^ which attract positive counterions to the solid–liquid interface, forming an electric double layer with a net positive charge density. An applied electric field of a few tens of volts exerts a body force on the double layer causing its motion, which is transferred by viscous drag to the neutral bulk fluid, thereby inducing a bulk flow.^10^ In contrast to pressure-driven flow, where a uniform driving force leads to a parabolic flow profile, in electro-osmotic flow the driving force is localized in the thin electric double layer near the solid surface, producing a nearly uniform velocity profile across a pore cross-section when the pore width *W* is a few times larger than the Debye length λ_D_. If the pore width *W* is so small that the double layers at two opposite pore walls overlap, different phenomena occur; and if *W* ≫ λ_D_, the flow remains confined close to the wall, with no effect on the bulk flow.

Electro-osmotic dewatering has been studied across a range of materials, including soils, sludges, and, more recently, food and biomass.^3,7,11–14^ The effects of applied voltage and initial moisture content on the drying kinetics have been mostly studied at the macroscopic scale, by measuring the mass of water removed as a function of time. Higher energy efficiencies per kilogram of water removed have been reported for electro-osmotic dewatering.^7^ Electrolyte concentration, ion valency, and molecular weight, as well as electrode configuration, spacing, and number, have also been shown to influence dewatering performance.^15–18^ Although these macroscopic, empirical relationships between operating conditions and overall water removal can help to optimize performance, they do not provide insights into the underlying pore-scale transport, which is crucial for rational process design.

A scaling analysis comparing the energy required for sustaining pressure-driven flow and electro-osmotic flow through a pore of width *W* shows that the volume-averaged viscous dissipation rate^19^ per unit volume (in W/m^3^) scales as ${ė}_{\mathrm{diss}}∼\mu \frac{u_{\infty }^{2}}{W^{2}}$ for pressure-driven flow and as ${ė}_{\mathrm{diss}}∼\mu \frac{u_{\infty }^{2}}{W\lambda_{D}}$ for electro-osmotic flow, where the characteristic velocity *u*_∞_ is taken to be the maximum velocity for both flow conditions, and μ is the shear viscosity.^10^ In typical conditions where λ_D_ < *W*, this analysis suggests that pressure-driven flow is more energy efficient than electro-osmotic flow for driving a flow with velocity *u*_∞_. To reconcile the seemingly contradictory scaling argument with experimental reports of higher energy efficiency for electro-osmotic dewatering, it is essential to investigate the pore-scale characteristics of the inhomogeneous flow field in the porous medium. The performance of electro-osmotic dewatering is indeed strongly influenced by the internal porous microstructure of the material: the porosity, the distribution of pore and pore-throat sizes, and the connectivity of the pore network. The effect of this microstructure on electro-osmotic transport remains poorly understood, in part because an experimentally validated, pore-scale framework is lacking.

Experimental studies of the microscale, hydrodynamic effects of electro-osmosis have focused on single microchannels.^20,21^ The pore-scale characteristics of electro-osmotic flow in a porous medium have been studied with numerical simulations, which however have not yet been validated experimentally.^22^ For instance, previous numerical simulations have neglected the dielectric properties of the solid phase, which can lead to an underestimation of electro-osmotic permeability, whereas assuming an ideal dielectric solid may result in an overestimation.^23^ Numerical studies have been used to compare the performance of electro-osmotic flow against the benchmark of pressure-driven flow in porous media^24,25^ by quantifying the velocity distributions in both cases. However, in these studies, lower velocity regions near solid walls have not been included in the computed velocity distributions. These low-velocity regions could affect dewatering performance as they can correspond to trapped water.

In this work, we develop an experimentally validated, pore-scale comparison of electro-osmotic and pressure-driven flow in a heterogeneous porous medium, to reveal how the internal porous microstructure influences the electro-osmotic flow velocity distribution. We compare electro-osmotic and pressure-driven flow under controlled experimental conditions in the same well-defined heterogeneous porous geometry using particle tracking velocimetry (PTV) to quantify the pore-scale velocity fields and validate a multiphysics model. To link microstructure to transport, we compare the probability density functions of the velocity for both flows, from both experiment and simulation. Because the model is validated against pore-scale measurements, it provides a framework that can be applied to other microstructures without new experiments, offering a basis toward the rational design and optimization of electro-osmotic dewatering.

## Experimental Methods

### Experimental Setup

A schematic of the experimental setup is shown in Figure 1a. An inverted optical microscope (IXplore IX73, Olympus) was used in combination with a CMOS camera (acA1920-150uc, Basler, 1920 × 1200 pixel sensor) and a 40× objective lens to capture images of the micromodel, with a resolution of 0.15 μm/px. The porous medium micromodel (2.3 mm × 7.75 mm) was designed to have a heterogeneous microstructure (Figure 1b), which consists of a random arrangement of cylinders with different radii.^26^ The random positioning and different radii of the cylinders create pore throats of different widths, with a probability density function that follows a power law.^27^ The porosity of the micromodel, defined as the ratio of the volume of voids to the total volume, was 0.40. The height of the micromodel was 20 μm. For electro-osmotic flow experiments, two platinum electrodes of 2 mm diameter (MW-4130, Basi Research Products) were inserted at the inlet and outlet of the micromodel and connected to a power generator (EDU33211A, Keysight Technologies) with an amplifier (PZD700A, TREK, Inc.) to apply a voltage of 100 V. For pressure-driven flow experiments, a syringe pump (Pump 11 Pico Plus Elite, Harvard Apparatus, Inc.) delivered water to the device through 1/16″ outer diameter and 0.5 mm inner diameter tubing. The water was delivered with a volumetric flow rate of *Q* ≈ 0.13 μL min^–1^.

**Figure 1:**
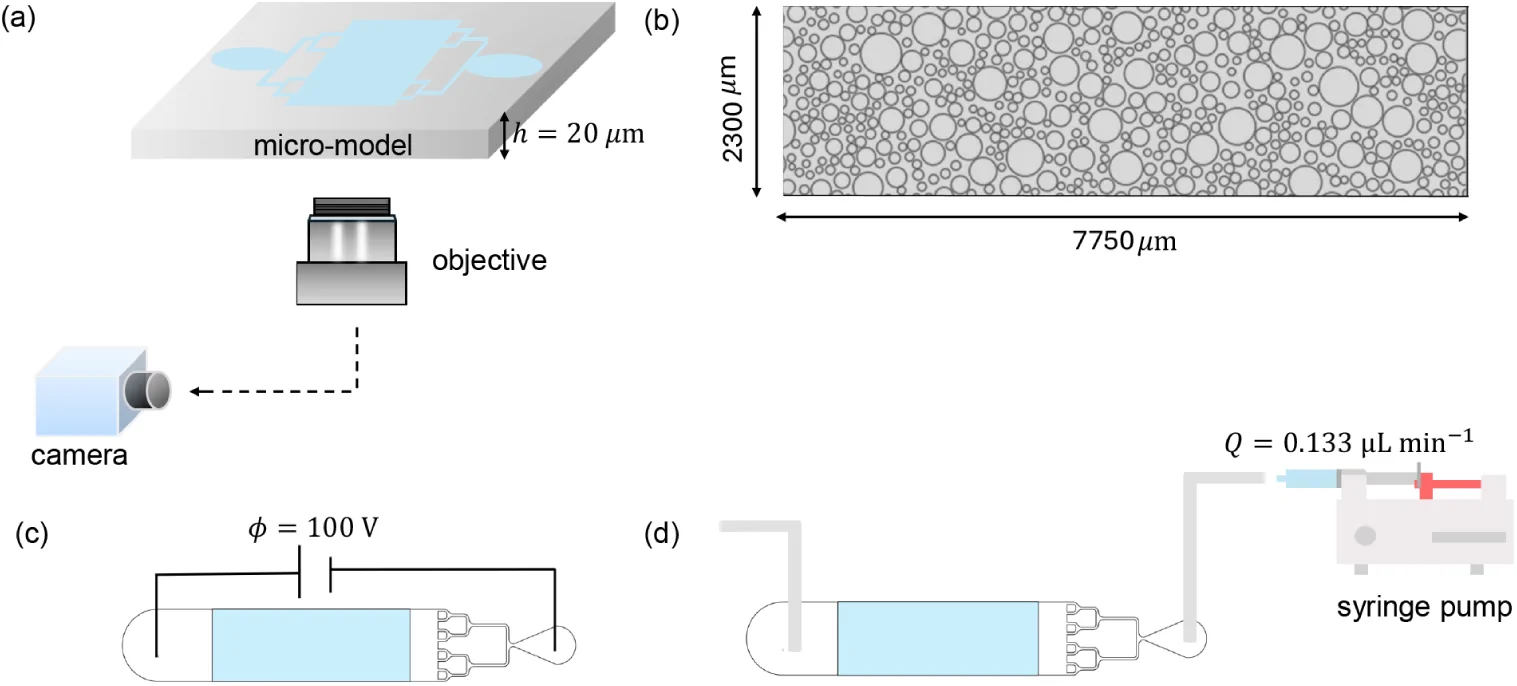
(a) Schematic of the experimental setup used with the PDMS micromodel. Water transport is recorded using an optical microscope equipped with a camera. (b) The micromodel consists of a random arrangement of cylindrical pillars, producing a heterogeneous pore structure. (c) Water transport driven by electro-osmosis is achieved by applying 100 V across two wire electrodes placed at the inlet and outlet of the micromodel. (d) Pressure-driven water transport is generated using a syringe pump delivering a volumetric flow rate of *Q* ≈ 0.133 μL min^–1^.

### Porous Micromodel Fabrication

The microfluidic device used in this study consists of a polydimethylsiloxane (PDMS) porous micromodel bonded to a glass slide. Porous matrices like soil and biomass present negative surface charges. PDMS acquires a negative surface charge through deprotonation of silanol groups upon treatment with oxygen plasma. Because the zeta potential of a charged surface is independent of the specific surface chemistry, the negatively charged PDMS surface is representative of a negatively charged porous matrix. The mold for the porous micromodel was fabricated by standard lithography methods on a silicon wafer. Subsequently, the micromodel was made by pouring a mixture of PDMS and a curing agent (SYLGARD 184 PDMS Elastomer, DOW Corning) with a 10:1 mass ratio onto the silicon wafer. Before pouring, the mixture was degassed in a desiccator to remove all bubbles. Afterward, the poured PDMS mixture was degassed again to remove bubbles and then cured at 70 °C in an oven for ∼3 h. Then, a circular 1.20 mm diameter inlet and outlet were punched into the micromodel. To ensure uniform properties across the entire micromodel, the bottom glass slide consists of a thin spin-coated layer of PDMS. After curing the PDMS layer in an oven, the coated slide and top part of the micromodel were treated with an oxygen plasma cleaner (PDC-001, Harrick Plasma) at the highest setting for 2 min and 20 s. Then the coated slide and micromodel were sealed to each other. Oxygen plasma treatment generates silanol groups on the PDMS surface, which deprotonate upon contact with water and are essential for establishing electro-osmotic flow. The micromodel was used immediately after plasma activation for EOF experiments, to ensure reproducible surface charge and hydrophilicity. Contact angle measurements showed that the hydrophilicity decays over time scales longer than 24 h; therefore, within the time frame of the experiments, the surface properties of PDMS did not change.

### Glass Microchannel Fabrication

The surface charge density and ionic concentration arising from wall deprotonation and trace salt naturally present in water are unknown parameters and therefore must be estimated through calibration. To enable this, a glass channel was fabricated and employed as a calibration platform. Glass and oxygen-plasma-treated PDMS possess similar surface chemistries, as both have silanol functional groups. Moreover, previous studies have demonstrated that plasma-treated PDMS and glass exhibit comparable electro-osmotic mobilities.^28^ This provides a sound justification for using a glass microchannel as a surrogate system for calibrating properties arising from the electrolyte–surface interface. By fitting the measured electro-osmotic flow in the glass channel with the model, values for surface charge density and ionic concentration were obtained. These values were then used as input parameters for simulations of electro-osmotic flow in the plasma-treated PDMS micromodel, based on the similarity of their silanol surface chemistries. The setup for the calibration experiments consists of two 3D-printed wells (Form3+, Formlabs) with a height of 30 mm, a width of 10 mm, and a length of 10 mm. Each well has a slot positioned 20 mm above the base to accommodate a borosilicate glass microchannel (30 mm in length, 2 mm in width, and 100 μm in height). Following insertion, the glass channel was fixed using a two-component epoxy adhesive (Bison Combi Snel Rapide). The well lid, designed to hold the electrodes, contains two openings: one for electrode insertion and a second for venting gaseous products generated during possible electrolysis. A plate electrode (stainless steel, 10 mm in length, 6 mm in width) was bonded to each lid using epoxy adhesive. The glass microchannel connecting the two wells enables observation of electro-osmotic flow induced by the applied electric field.

### Flow Visualization

To visualize the flow, an aqueous suspension of near-neutrally buoyant polystyrene microspheres (Sulfate Latex Beads, 8% w/v, 1 μm, Thermo Fisher Scientific) prepared in Milli-Q water was injected into the PDMS micromodel to perform particle tracking velocimetry (PTV). The concentration of the suspension for the electro-osmotic flow (EOF) experiments was 1.7 × 10^10^ particles mL^–1^. For these experiments, the suspension was manually introduced in the micromodel using 1/16″ outer diameter, 0.5 mm inner diameter tubing connected to a syringe. Platinum electrodes were then inserted at the inlet and outlet. After allowing sufficient time for any residual pressure-driven flow to decay after the injection, a potential of 100 V was applied. This field strength is consistent with values commonly used in experimental microfluidic studies of electro-osmotic flow.^29^ Such fields are needed to generate particle displacements large enough to be measured by PTV within the short observation window of 3 s used to limit Joule heating. Joule heating in electro-osmotic flow was characterized by Xuan et al., who observed a temperature rise over ∼15 s using a buffer with a conductivity of 0.21 S/m.^30^ In our experiments, the fluid is Milli-Q water, in which the only ions originate from surface deprotonation, giving a conductivity several orders of magnitude lower.^31^ Because Joule heating scales with conductivity (at a given applied potential), the heating in our system is negligible over 3 s. Furthermore, the flow was steady throughout the 3 s recording, confirming that the flow was fully developed during the measurements. Gas evolution can occur at the electrodes over the time scale of the experiments. However, in this configuration no macroscopic bubbles were observed, and no associated pressure buildup or perturbation of the electro-osmotic flow was detected. The experiment was recorded with a frame rate of 100 s^–1^.

For the calibration experiment, a suspension of polystyrene microparticles (Sulfate Latex Beads, 8% w/v, 1 μm, Thermo Fisher Scientific) in Milli-Q water was introduced into the wells and allowed to fill the microchannels. After the particles exhibited negligible convective motion compared to Brownian motion, electric field strengths of 50, 80, 100, 120, and 150 V cm^–1^ were applied across the channel to induce electro-osmotic flow. The experiment was recorded for 3 s with a frame rate of 20 s^–1^

It is important to note that in these experiments, the particles used in PTV are not ideal passive tracers. Because they are negatively charged with a zeta potential ζ_particle_ = (−31 ± 6) mV, they undergo electrophoresis in the externally applied electric field. The electrophoretic motion is toward the negative electrode, in the opposite direction to the electro-osmotic flow. The electrophoretic velocity **v**_ep_ = (ϵζ_particle_/μ)**E** is proportional to the local electric field **E** and depends on the electric permittivity ϵ and viscosity μ of the fluid (in this case water). Similarly, the electro-osmotic flow velocity **v**_eo_ = (ϵζ_PDMS_/μ)**E** is proportional to the local electric field and the zeta potential ζ_PDMS_ of the PDMS micromodel.^10^ The observed particle velocity is a superposition of both effects. Because both velocities are linearly dependent on the local electric field, statistically the normalized velocity PDF of electro-osmotic flow is not strongly influenced by the presence of electrophoretic motion.

The same particle suspension with a concentration of 1.7 × 10^13^ particles mL^–1^ was used for the pressure-driven flow experiments at a constant flow rate of *Q* = 0.133 μL min^–1^. This volumetric flow rate was selected to optimize the particle tracking conditions in our setup. Once steady-state conditions were established, the flow was recorded for approximately 10 s with a frame rate of 70 s^–1^.

A small region of the microfluidic micromodel is recorded for further analysis in both pressure-driven flow and electro-osmotic flow experiments. The recorded area is shown in Figure 2a. Figure 2b displays an image stack in one of the electro-osmotic flow experiments, where particle trajectories over time are visible.

**Figure 2:**
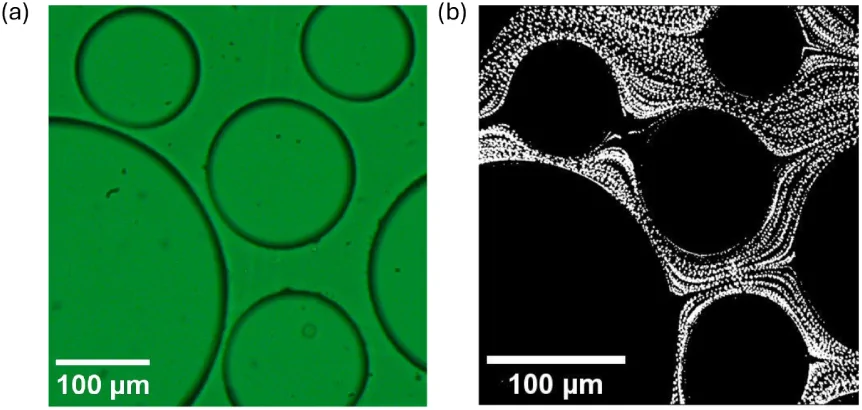
(a) Region of the micromodel recorded during both electro-osmotic and pressure-driven flow experiments. (b) The same region, showing an experimental image stack in which particle trajectories over time are visible in an electro-osmotic flow experiment.

### Image Processing and Particle-Tracking Velocimetry

Image processing was performed in Fiji (ImageJ) prior to particle detection for the PTV. To remove the static background and fixed noise, an average background image was first generated from the image sequence and subtracted from each frame. This background correction step enhances the contrast of moving particles while suppressing stationary features. The resulting images were then converted to binary masks by applying a threshold, isolating particles. The binary images were subjected to morphological operations including dilation, and erosion to produce smooth circular particles suitable for subsequent particle detection. Particle detection was performed in MATLAB using the regionprops­() subroutine to identify particle coordinates used in the tracking algorithm. These particle coordinates serve as input for the tracking algorithm to find particle trajectories and determine their velocities.

Particle trajectories were obtained using the V-trackMat algorithm.^32^ The algorithm uses a nearest-neighbor approach based on the ipdm routine and relies on the assumption that the frame rate is high enough to ensure that particles do not jump farther than the average interparticle distance. For each pair of consecutive frames, particle positions were first projected using the velocity computed in the previous frame, and then the corresponding particles were identified within a search window defined by the average interparticle distance around the projected positions. To prevent oversampling biases associated with low particle velocities and undersampling biases associated with high particle velocities in the probability density function (PDF), all trajectories were interpolated to contain an equal number of spatial points per unit length.

## Numerical Simulations

### Electro-Osmotic Flow

To model EOF, a coupling between ionic transport, electrostatics and hydrodynamics is required. This is done by simultaneously solving the Nernst–Planck equation, Gauss’s law, and the Navier–Stokes equations. The Nernst–Planck equation governs the transport of ionic species under the influence of diffusion, convection, and an electric field:

1 $$\frac{\partial c_{i}}{\partial t}=-\nabla \cdot J_{i}$$

2 $$J_{i}=-D_{i}\nabla c_{i}-\frac{D_{i}z_{i}e}{k_{B}T}c_{i}\nabla \phi +c_{i}u$$

where ***J***_*i*_ is the flux and *c*_*i*_ the concentration of ion *i*, $D_{i}$ its diffusion coefficient, *z*_*i*_ its valence, *e* the elementary charge, ϕ the electrostatic potential, *k*_B_ Boltzmann’s constant, *T* the absolute temperature, and ***u*** the fluid velocity.

The electrostatic potential is determined by Gauss’s Law:

3 $$\nabla^{2}\phi =-\frac{\rho_{e}}{\epsilon }$$

where ρ_e_ = ∑_*i*_ *z*_*i*_ *ec*_*i*_ is the net charge density and ϵ is the permittivity. This formulation ensures that the ionic distribution from the Nernst–Planck equation generates the potential difference responsible for the electro-osmotic driving force.

Finally, the fluid flow induced by the body force of the electric field on the mobile charges is described by the incompressible Navier–Stokes equations, with the electric body force −ρ_e_∇ϕ:

4 $$\rho \left(\frac{\partial u}{\partial t}+u\cdot \nabla u\right)=-\nabla p+\mu \nabla^{2}u-\rho_{e}\nabla \phi$$

5 ∇·u=0

where ρ is the fluid density, μ is the fluid viscosity, and *p* is the fluid pressure. Note that for an incompressible flow the pressure *p* appears as a Lagrange multiplier to impose the incompressibility constraint given by eq 5. Eqs 1–5 are subject to the following boundary conditions. An electric potential was applied at both the inlet and outlet. We note that the experimental and numerical results are compared through the velocity PDF normalized by the mean velocity. Because the electro-osmotic velocity is linearly proportional to the applied field, changing the applied potential rescales the velocity by a constant factor and leaves the normalized distribution unchanged. The comparison therefore does not depend on the experimental and simulated potentials being identical. The obstructions were assigned the dielectric constant of PDMS, while the fluid domain was assigned the dielectric constant of water. In the experiments, deionized water was used and no additional salts were introduced. Therefore, in the simulations, only monovalent ions originating from the deprotonation of the PDMS surface, with ionic diffusivities on the order of 10^–9^ m^2^ s^–1^, typical for ions in aqueous solution, were assumed. Rather than resolving each ionic species separately, the solution was modeled as an effective monovalent electrolyte, with the concentration obtained from the calibration described below. This effective concentration accounts for all ions present in the deionized water, including the counterions from surface deprotonation and the H^+^ and OH^–^ from water autoionization, as it is fitted to the measured electro-osmotic flow rather than calculated from the individual ion concentrations. Charge neutrality was imposed by including positive and negative monovalent species of equal concentration (*z* = +1, −1), so that the net charge density is zero in the bulk and nonzero only within the electric double layer near the charged surfaces. No-flux boundary conditions were imposed at the bottom and top walls as well as on the surfaces of the obstructions. A zero-gauge pressure was imposed at both the inlet (P1) and the outlet (P2), and the channel’s top and bottom walls and surfaces of obstructions were subjected to no-slip boundary conditions. Finally, a surface charge density σ was assigned to the walls and surfaces of the obstructions. These boundary conditions are summarized in Figure 3.

**Figure 3:**
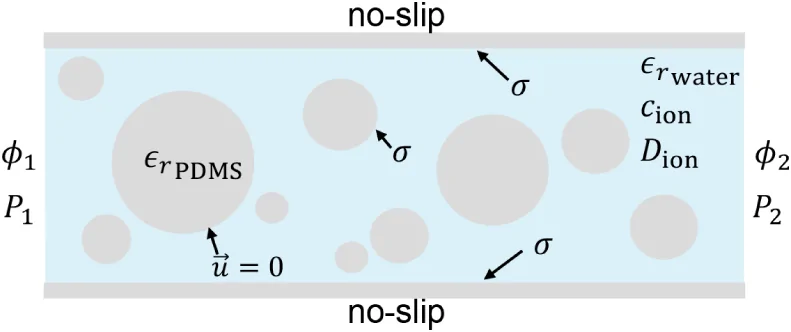
Schematic of the boundary conditions and parameters used in the simulation for electro-osmotic flow and pressure-driven flow. For electro-osmotic flow *P*_1_ = *P*_2_ = 0, while fore pressure-driven flow ϕ_1_ = ϕ_2_ = 0.

Using the height of the microchannel (20 μm), a mean velocity of electro-osmotic flow in the order of *u* = 1 × 10^–4^ m s^–1^, together with the density (ρ = 1000 kg m^–3^) and viscosity (μ = 1 × 10^–3^ Pa s^–1^), the Reynolds number was estimated to be *Re* ≈ 2 × 10^–3^. Given this very low Reynolds number, inertial effects are negligible, and the flow can be accurately described using the Stokes approximation. Accordingly, the inertial terms in eq 4 are dropped, and the flow is governed by the Stokes equations, 0 = −∇*p* + μ∇^2^ ***u*** – ρ_e_∇ϕ, ∇· ***u*** = 0, solved together with the boundary conditions described above.

On the basis of the governing equations and boundary conditions, two-dimensional steady-state electro-osmotic flow was simulated using the same geometry as the experimental micromodel. The governing equations were solved using COMSOL Multiphysics (version 6.2), which returns the electric field, velocity field, pressure field, and energy dissipation rate. The numerical method was validated against previous simulation results by Fraia et al.^33^ for a homogeneous structure.

### Calibration of Model Parameters

The surface charge density and the ionic concentration were determined concurrently by fitting the numerical model to the experimental data for the case of flow through a simple rectangular geometry. Electro-osmotic flow experiments were performed in a rectangular glass microchannel, where the flow velocity was measured under various applied electric fields. The numerical simulation, performed using an identical rectangular geometry with the same dimensions, was then fitted to the experimental measurements by simultaneously adjusting the surface charge density and the initial ionic concentration. The fit was performed against the measured particle velocities without separately correcting for the electrophoretic contribution; the calibrated surface charge density is therefore an apparent value that already incorporates the electrophoretic offset. As a result, when these parameters are applied to the micromodel, the simulation reproduces the observed velocity, so that the model and experiment are consistent. This calibration procedure yielded an ionic concentration of *c*_*i*_ = 5 × 10^–7^ mol L^–1^ and a surface charge density of σ = 5 × 10^–5^ C m^–2^. These ionic concentration and surface charge density are in good agreement with the literature.^28^

The calibrated parameters obtained from the rectangular channel are then applied to the simulations in the porous micromodel. The electro-osmotic flow is computed in a domain discretized using a mesh with approximately 1 × 10^6^ elements. The element size decreases to a minimum of ∼0.5 μm closer to the boundaries, so that the boundary layers are well-resolved. The values of the boundary conditions and parameters can be found in Table 1.

**Table 1:** Parameters and Their Values Used in the Numerical Simulation

parameter	Symbol	Value	Unit
Diffusivity	$D_{ion,1}$ , $D_{ion,2}$	10^–9^	m^2^ s^–1^
Initial ion concentration	*c*_1_, *c*_2_	5 × 10^–7^ mol L^–1^	mol L^–1^
Applied potential	ϕ_1_, ϕ_2_	0, 30	V
Water dielectric constant	ϵ_water_	80	-
PDMS dielectric constant	ϵ_PDMS_	3	-
Species charge	*z*_1_, *z*_2_	+1, −1	-

In order to determine the velocity PDF of the simulated EOF in the porous micromodel, the absolute velocity was extracted from the velocity field. To prevent sampling bias, velocities were sampled on a uniform square grid with a spatial resolution of 2 μm. The grid size was selected after performing a convergence study by checking that the velocity PDF was not changing.

### Pressure-Driven Flow

Pressure-driven flow is simulated in the same geometry as electro-osmotic flow. The incompressible Navier–Stokes eq 4 was solved, only now without an electric body force and with different boundary conditions. At the inlet channel, an inlet velocity was imposed, while the outlet was defined as a pressure boundary with zero-gauge pressure. The flow field was computed using a stationary solver, and the mesh was refined near the walls to resolve boundary layers (minimum element size of ∼0.5 μm) and ensure accurate velocity profiles in the pores. The domain was discretized using a mesh with approximately 10^6^ elements. As for the electro-osmotic flow, the PDF of absolute velocities in the simulated velocity field was determined for direct comparison with experimental measurements.

## Results and Discussion

Figure 4a shows the magnitude of the electric field. Figure 4b and 4c show the magnitude of velocity observed in the simulation of electro-osmotic flow and pressure-driven flow, respectively. A subdomain of the entire simulation is shown here. Unlike the velocity field in regular structures, the velocity field in the irregular structure is heterogeneous. This is caused by a combination of the dielectric property of the obstructions, their size, and placement. Due to the dielectric property of the obstructions, the electric field lines near the surface become more curved. This leads to an increase of the tangential component of the electric field along the surface. Due to the difference in size and placement of the obstructions, the distortions in the electric field lines will hence vary in the entire structure. Because the electro-osmotic flow velocity is dependent on the electric field strength, this will lead to a heterogeneous velocity profile. As expected, we observe that in narrow constrictions the velocity is highest. This arises primarily from local enhancement of the electric field. By current continuity, the field lines are concentrated where the cross-section narrows, increasing the local field magnitude. Since the electro-osmotic flow velocity is proportional to the local field, the velocity increases accordingly. It can also be observed that channels perpendicular to the electric field have low velocities, even when the channel is narrow. This can be explained by the fact that a reduced electric field is present in the direction required to have a flow in a perpendicular channel. On the contrary, lower velocities can be found in larger pore bodies (orange and yellow regions). This is because the electric field is weaker there. As the cross-section widens, the field lines spread out and the local field magnitude decreases, reducing the electro-osmotic velocity. As explained before, the electro-osmotic flow profile is typically flat. This profile corresponds to the velocity profile between the two obstructions on the black line in Figure 4g. However, in an irregular structure, velocity profiles deviate from this typical profile. This is exemplified in Figure 4h and Figure 4i, which correspond to the profiles in the pore throats on the red and green lines, respectively. Although no overall (macroscopic) pressure gradient is applied at the inlet and outlet, this phenomenon can be explained by the presence of local pressure gradients. This is due to the heterogeneous nature of the structure, wherein multiple pores with different flow velocities are connected (due to varying electric field as a result of the inhomogeneity of the structure). The difference in velocities in connecting pore bodies, causes a pressure difference between neighboring bodies. The pore-to-pore local pressure difference hence creates a secondary driving force for fluid flow that causes deviations from the typical electro-osmotic flow profile expected for a straight channel. The simulated pressure field for the electro-osmotic flow is shown in Figure 5. The velocity vectors further reveal that the flow profile within a pore is influenced by the direction of the incoming flow, which is governed by the arrangement of the surrounding obstructions and pore throats. Figure 5b–d shows the velocity vectors corresponding to the same locations as in Figure 4b and c. As illustrated in Figure 5d, the incoming flow between the two obstructions arrives at an angle. While the electro-osmotic flow redirects it more closely along the pore throat axis, the overall flow orientation remains influenced by the direction of the incoming flow.

**Figure 4:**
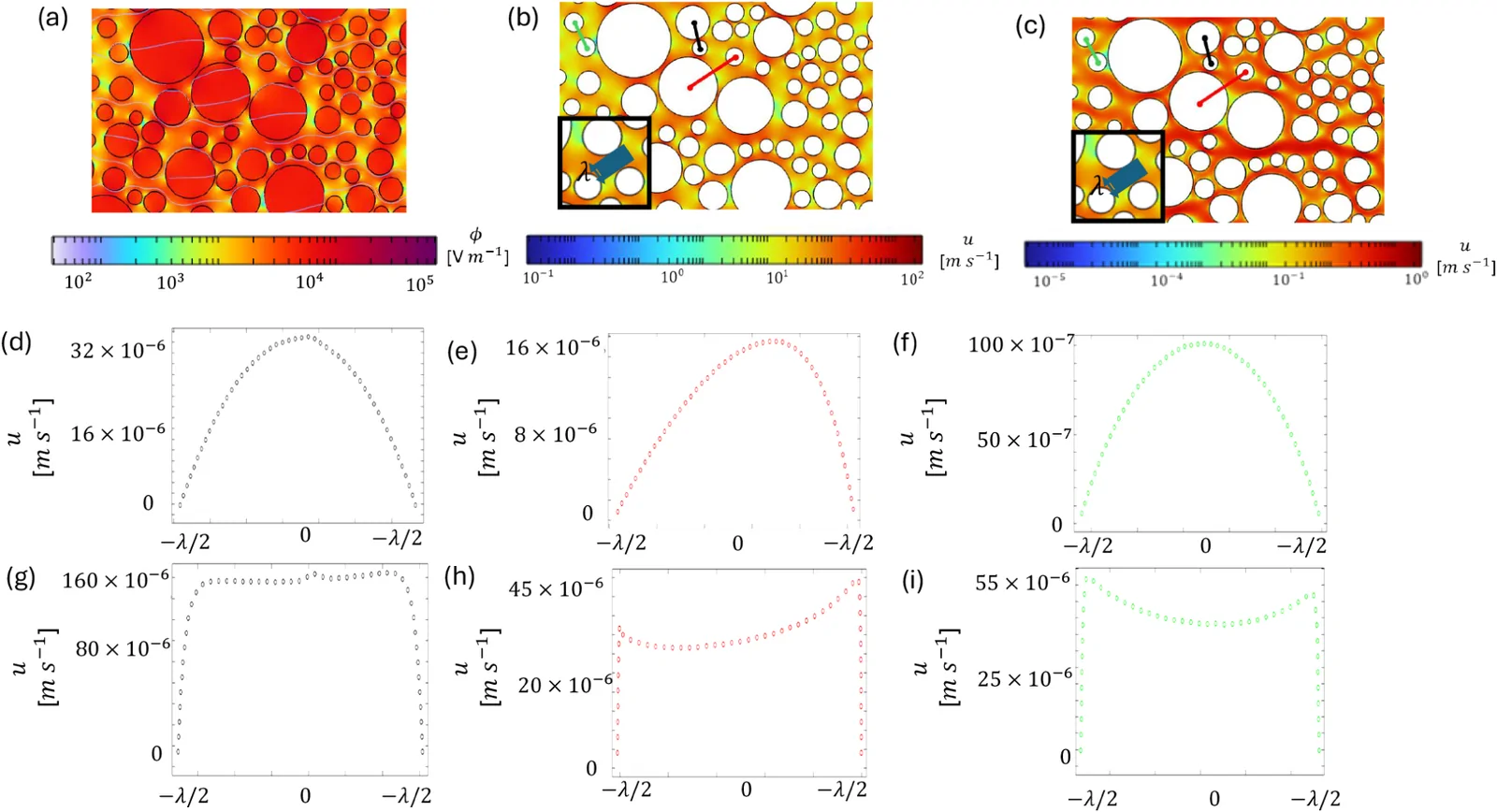
(a) Electric field strength magnitude within a selected region of the micromodel. (b) Electro-osmotic flow velocity magnitude within the same selected region of the micromodel. Velocity profiles at three representative locations are shown in Figure 4g-i. The corresponding positions are indicated by green, black, and red lines. (c) Pressure-driven flow velocity magnitude within a selected region of the micromodel. Velocity profiles at three representative locations are shown in Figure 4d-f, with the locations likewise indicated by green, black, and red lines.

**Figure 5:**
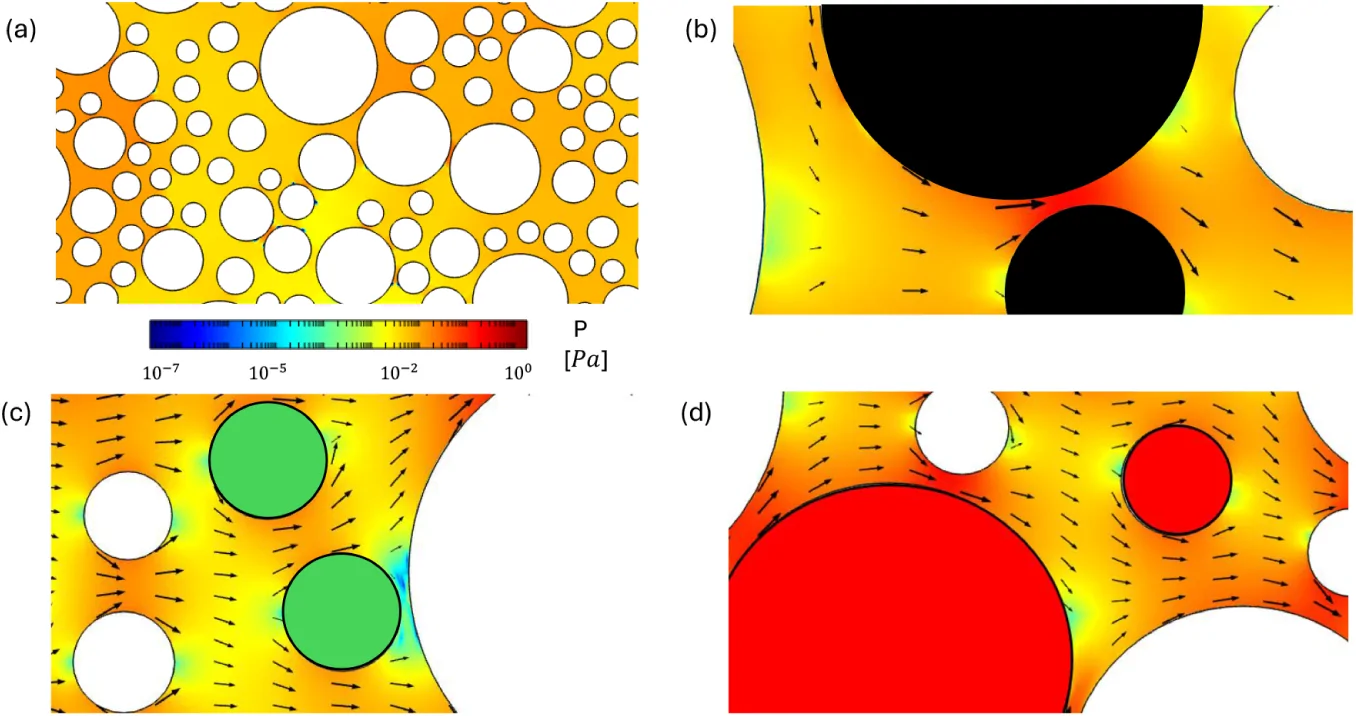
(a) Simulated pressure field for the electro-osmotic flow case. Local pressure differences develop between neighboring pores. (b,c,d) Velocity vectors in the pore throats, at the same locations as in Figure 4, showing that the flow within each pore is influenced by the direction of the incoming flow, set by the surrounding obstructions and pore throats. The arrows indicate flow direction only and are not scaled by velocity magnitude.

Contrary to the electro-osmotic flow case, it can be observed that pressure-driven flow leads to preferential flow paths (Figure 4c), caused by the high hydraulic resistance of narrow channels and low hydraulic resistance of wide channels. Turning to the velocity distribution within the channels, the case of pressure-driven flow typically shows parabolic shapes as expected.^34^

Figure 6 shows the probability density function of the velocity magnitudes observed in electro-osmotic flow and pressure-driven flow in the structure for both the simulation and the experiment, normalized by their mean velocities. The PDF of pressure-driven flow in a similar heterogeneous structure shows a similar power-law behavior to that reported by de Anna et al.^27^

**Figure 6:**
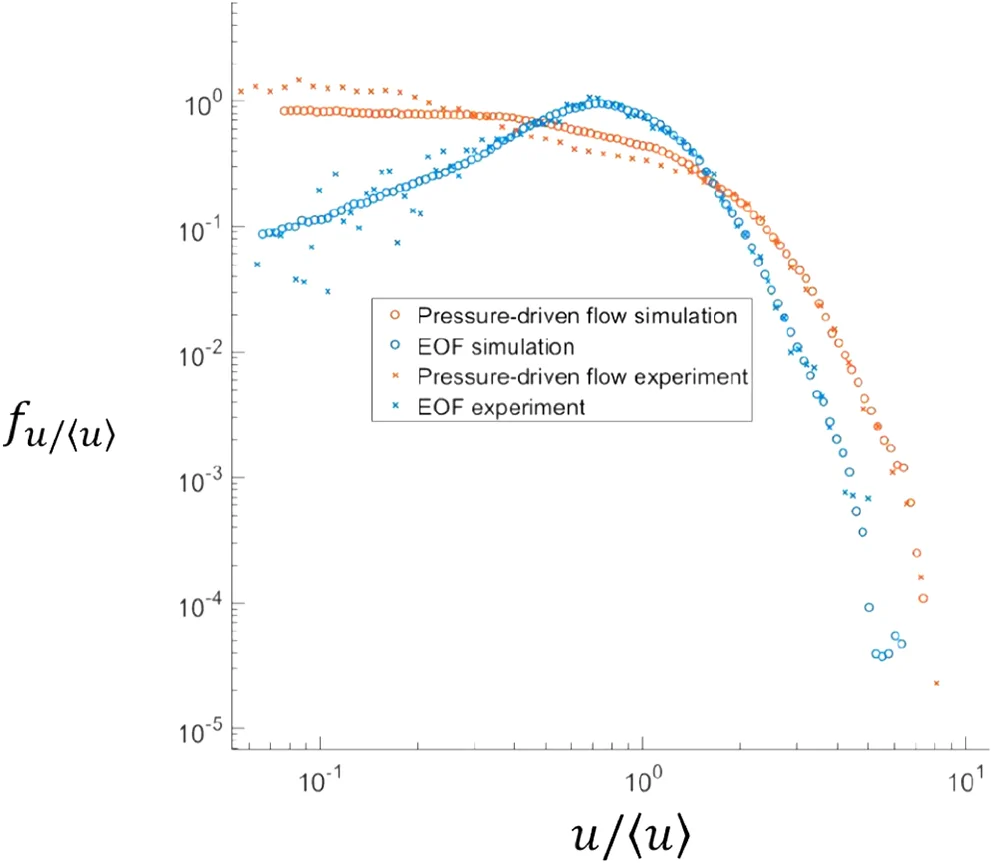
Probability density functions of velocity magnitudes normalized by the respective mean velocities for pressure-driven and electro-osmotic flows, where orange circles and crosses represent simulations and experiments of pressure-driven flow, respectively, and blue circles and crosses represent simulations and experiments of electro-osmotic flow, respectively.

For pressure-driven flow, the velocity probability density function obtained from the numerical simulations closely matches the experimentally measured distribution, and a good agreement is observed between simulation and experiment for electro-osmotic flow as well. In comparison to pressure-driven flow, electro-osmotic flow shows a lower probability for normalized velocities ranging from 0.1 to 1. This lower probability of both very low and very high normalized velocities is a consequence of the almost uniform, plug-like velocity profile of electro-osmotic flow, which inherently produces less variability in velocity. The plug-like profile is characterized by the peak at the mean velocity and does not exhibit velocity extremes, even in highly heterogeneous pore structures. Figure 6 shows that pressure-driven flow has a higher probability for high normalized velocities in the range of 2–10. Pressure-driven flow produces preferential flow paths and stagnant zones that amplify fast and slow regions (see Figure 4c). Besides, the parabolic profiles within single pores also possess a broader velocity distribution.^27^ These two factors together produce the wider velocity distribution for pressure-driven flow. For the same volumetric flow rate, the viscous dissipation for electro-osmotic flow is 1.8 × 10^–15^ W and for pressure-driven flow 5.1 × 10^–17^ W, consistent with the scaling argument given in the Introduction. We hypothesize that because electro-osmotic flow leaves less stagnant water, as shown by the analysis below, dewatering by EOF can still be more efficient despite this higher energy dissipation to sustain the flow.

In order to get a better understanding of where water would be stagnant or trapped and where water is more easily moved, low-velocity regions and high-velocity regions are extracted from the simulated surface velocity plot. The low-velocity regions are defined here as all velocities in the range of zero to one-quarter of the mean velocity. The high-velocity regions are defined as all velocities larger than the mean velocity. In the case of electro-osmotic flow, the low-velocity regions (Figure 7a) are very patchy and localized. Furthermore, the low velocities are mainly present in the pore throats perpendicular to the applied electric field. In pressure-driven flow, the low-velocity regions consist of larger connected clusters (Figure 7b). To quantify this difference, we analyzed the low-velocity regions in both cases. For electro-osmotic flow, the stagnant regions amount to approximately 1100 small isolated patches, whereas pressure-driven flow consists of 17 large connected clusters. Despite this much larger number of regions, the total stagnant area is smaller for electro-osmotic flow (28%) than for pressure-driven flow (48%). This means that EOF has a narrower residence time distribution than in pressure-driven flow, because in pressure-driven flow, the fluid has a higher chance to be trapped in stagnant zones. Comparing the high-velocity regions of the two driving forces, it is apparent that in the case of electro-osmotic flow, the high velocities are more spread over the entire structure (Figure 7c) in pores that are parallel to the applied field. In pressure-driven flow, a few pathways exist where the water flow is fast (Figure 7d).

**Figure 7:**
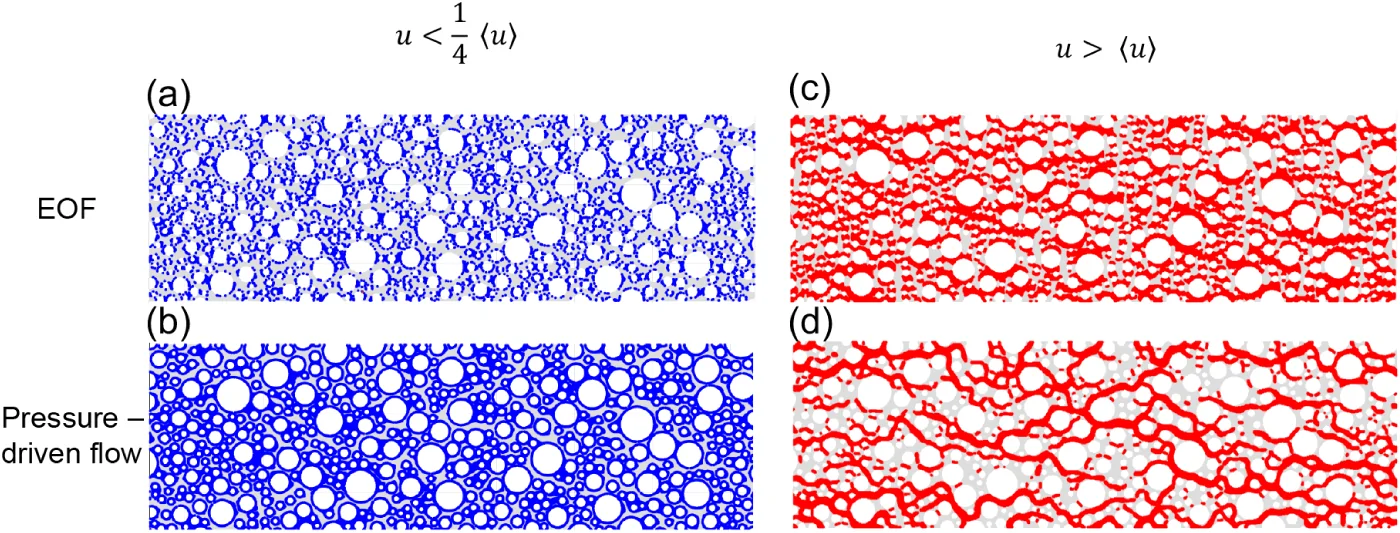
Low- and high-velocity regions in the electro-osmotic and pressure-driven flow simulations over the entire micromodel are shown. Low-velocity regions $\left(u<\frac{1}{4}\left\langle u\right\rangle \right)$ for electro-osmotic and pressure-driven flows are shown in blue in panels (a) and (b), respectively, while high-velocity regions (*u* > ⟨*u*⟩) are shown in red in panels (c) and (d), respectively.

The narrower velocity distribution (Figure 6) and the more even spatial distribution of high-velocity regions, with smaller stagnant patches (Figure 7), suggest that electro-osmotic flow should achieve more uniform dewatering in a heterogeneous microstructure than pressure-driven flow, for which a few high-velocity regions leave large connected stagnant zones. Although our experiments and simulations focus on steady flow through the porous microstructure, rather than dewatering, these observations shed light on the role of the microstructure in making EOF more energy-efficient per unit of removed water despite the higher energy dissipation for sustaining the flow.

## Conclusions

In this study, electro-osmotic flow and pressure-driven flow in a well-defined heterogeneous porous micromodel were investigated experimentally and numerically. Particle tracking velocimetry was used to measure the velocity at the pore scale. A multiphysics model was employed to simulate electro-osmotic flow and pressure-driven flow. The experimentally determined velocity probability density functions for both pressure-driven and electro-osmotic flow validated the numerical model. Pressure-driven flow exhibited a broad velocity distribution, characterized by preferential flow paths and stagnant zones. Electro-osmotic flow produced a narrower distribution with a peak at the mean velocity. This reflects the typical plug flow of electro-osmotic transport and exists even in highly heterogeneous porous structures despite the presence of locally induced pressure gradients. Besides, lower probabilities for lower velocities are found for electro-osmotic flow. This suggests that electro-osmotic flow can reduce stagnant zones. Analysis of low- and high-velocity regions revealed that electro-osmotic flow leads to a more uniform removal of water across the porous domain, whereas pressure-driven flow concentrates transport along a limited number of high-velocity pathways and leaves large regions of low mobility. Overall, this work reveals a pore-scale mechanism by which electro-osmotic flow acts as an effective transport mechanism in heterogeneous porous media. Because the multiphysics model reproduces the measured pore-scale velocity distributions, it can be applied to other geometries without further experiments. The velocity PDF and the spatial distribution of low- and high-velocity regions then serve as quantitative performance metrics that can be evaluated for different pore structures, porosities, and applied field strengths, allowing the conditions that minimize stagnant zones to be identified. The validated framework thus offers a basis toward the rational design and optimization of electro-osmotic dewatering as a function of the microstructure. The main limitation of the present study is that the micromodel and simulations represent a rigid, fully saturated structure, whereas real materials shrink continuously as water is removed. These geometric changes are coupled to the electrokinetics. Shrinkage alters the local pore widths and hence the electric field distribution and the electro-osmotic flow. The decreasing moisture content simultaneously lowers the electrical conductivity and increases the voltage drop across the material. This locally intensifies the field. Our static, fully saturated micromodel deliberately isolates the effect of microstructure on flow under controlled conditions, but it does not capture this evolving coupling between deformation and transport. A further simplification is the use of deionized water without an added background electrolyte, so that the only ionic species present arise from deprotonation of the surface silanol groups. This provides a minimal well-defined system suited to validating the framework but does not represent the salt-containing pore water of real applications. Future work should therefore extend the validated framework to deforming geometries so that the progressive shrinkage and the associated changes in electric field and flow can be modeled together over the course of dewatering. Finally, the background electrolyte is already included in the present framework as an input variable in the numerical model and can therefore be incorporated directly in future work.

The validated modeling approach presented here offers a predictive framework for assessing electro-osmotic performance in different complex and realistic biomass geometries, thereby supporting the rational design of electro-osmotic dewatering.

## #

Aix-Marseille Université, CNRS, Centrale Méditerranée, IRPHE, UMR 7342, 13384 Marseille, France
